# Mutation of LRP1 in cardiac neural crest cells causes congenital heart defects by perturbing outflow lengthening

**DOI:** 10.1038/s42003-020-1035-9

**Published:** 2020-06-16

**Authors:** Jiuann-Huey I. Lin, Timothy N. Feinstein, Anupma Jha, Jacob T. McCleary, Juan Xu, Angelo B. Arrigo, Grace Rong, Lindsey M. Maclay, Taylor Ridge, XinXiu Xu, Cecilia W. Lo

**Affiliations:** 1 Department of Critical Care Medicine, University of Pittsburgh, Pittsburgh, PA USA; 2 Department of Developmental Biology, University of Pittsburgh, Pittsburgh, PA USA; 3 School of Pharmacy, University of Pittsburgh, Pittsburgh, PA USA; 4 Department of Biological Sciences, University of Pittsburgh, Pittsburgh, PA USA; 5 Department of Neurosciences, University of Pittsburgh, Pittsburgh, PA USA

**Keywords:** Embryology, Disease model

## Abstract

The recent recovery of mutations in vesicular trafficking genes causing congenital heart disease (CHD) revealed an unexpected role for the endocytic pathway. We now show that mice with a C4232R missense mutation in Low density lipoprotein receptor related protein 1 (LRP1) exhibit atrioventricular septal defects with double outlet right ventricle. *Lrp1*^*m/m*^ mice exhibit shortened outflow tracts (OFT) and dysmorphic hypocellular cushions with reduced proliferation and increased apoptosis. *Lrp1*^*m/m*^ embryonic fibroblasts show decreased cell motility and focal adhesion turnover associated with retention of mutant LRP1 in endoplasmic reticulum and reduced LRP1 expression. Conditional deletion of *Lrp1* in cardiac neural crest cells (CNC) replicates the full CHD phenotype. Cushion explants showed defective cell migration, with gene expression analysis indicating perturbation of Wnt and other signaling pathways. Thus, LRP1 function in CNCs is required for normal OFT development with other cell lineages along the CNC migratory path playing a supporting role.

## Abstract (web summary)

Lin et al. find that mutation in endocytic trafficking protein Lrp1 causes congenital heart defects in mice due to a requirement for Lrp1 in the neural crest lineage, where it regulates outflow tract lengthening. This study provides insights into how Lrp1 and the neural crest contribute to heart development.

## Introduction

Congenital heart disease affects nearly 1% of neonates per year in the United States^1^ and is the leading cause of neonatal death^2^. With improved survival made possible by advances in critical care and surgical palliation, there are now over 2 million children and adults with CHD in the United States^3^. However, surviving CHD adults continue to face medical challenges, including rehospitalization, surgical re-intervention, or even need for heart transplantation. The development of better clinical management strategies will require a better understanding of the developmental etiology and pathomechanisms of CHD.

An unexpected role for the endocytic pathway in CHD^4^ pathogenesis was revealed by a large-scale mouse forward genetic screen for mutations causing CHD^4^. Among these was a missense mutation in *Lrp1*, a member of the multifunctional low-density lipoprotein receptor-related protein (LRP) family^5,6^. The LRP1 precursor is a 600 kD protein that is cleaved in the trans-Golgi network, generating a 515 kD α-chain (amino acid 20–3944) and a membrane bound 85 kD β-chain (amino acid 3945–4545). The α-chain of LRP1 contains various repeated motifs and the β-chain contains the C4232R mutation and two cytoplasmic NPxY sequences^5^ that serve as signals for endocytosis^5,6^. LRP1 was originally described to mediate endocytosis of lipoproteins, but it is now also appreciated that LRP1 can bind dozens of other ligands. This includes many different transmembrane receptors—LRP1 modulates their activity and downstream intracellular signaling, including their internalization, sorting to different endosomal compartments, recycling, and their degradation^5,6^.

Mice homozygous for a missense mutation in the cytoplasmic domain of the LRP1 β-chain (*Lrp1*^*m/m*^) survive to mid-gestation, dying prenatally from structural heart defects^4^. This stands in marked contrast to the peri-implantation lethality of *Lrp1* knockout (KO) mice^7^. This *Lrp1*^*C4232R*^ (*Lrp1*^*m/m*^) mouse model reveals a role for *Lrp1* in CHD pathogenesis. Using this unique *Lrp1*^*m/m*^ missense mutant and conditional KO mouse models with a floxed allele of *Lrp1* (*Lrp1*^*f/f*^) crossed with different Cre drivers, we now investigate the pathomechanism for CHD arising from *Lrp1* perturbation. Our studies indicate an essential role for *Lrp1* in the cardiac neural crest (CNC) lineage, and a non-cell autonomous role in other cell lineages along the NCC migratory path. Further experiments with fibroblasts derived from *Lrp1*^*m/m*^ mice suggest that this likely involves LRP1 regulation of cell motility and cell signaling required for proper NCC targeting to the heart.

## Results

**Fig. 1:**
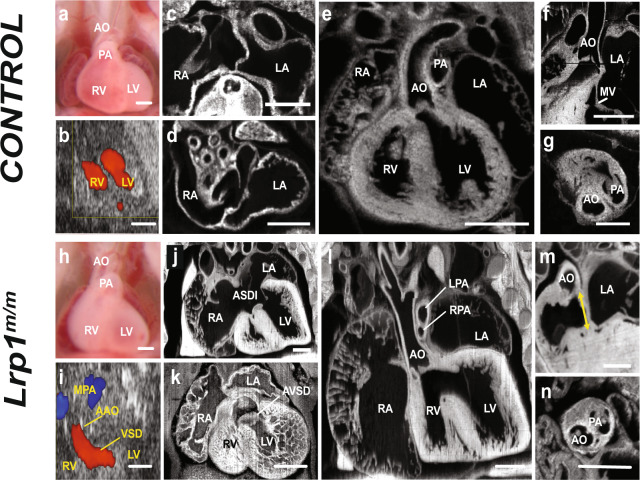
Homozygous *Lrp1*^*m/m*^ mutants exhibit a spectrum of cardiac defects. Compared with E14.5 normal control (**a**–**g**), *Lrp1*^*m/m*^ mutant embryo showed side-by-side great arteries (**h**, **i**, **l**, **n**). Echocardiogram using Vevo2100 of control embryo indicated intact ventricular septum with no evidence of interventricular shunt (**b**), which was confirmed by histopathology with ECM (**e**). Representative fetal ultrasound of the *Lrp1*^*m/m*^ mutant (**i**) showed a VSD and side-by-side great vessels with aorta positioned to the right of the pulmonary artery. This was also confirmed by ECM (**l**), which showed double outflow right ventricle with both the aorta (AO) and pulmonary artery (LPA and RPA) arise from RV and a large VSD. Compared to the wild-type control (**c**, **d**), the homozygous *Lrp1*^*m/m*^ mutant exhibited ASD primum (ASDI) (**j**), a part of AVSD, and ventricular part defect of AVSD (**k**). While there is aorta to mitral valve continuity in controls (**f**), the *Lrp1*^*m/m*^ mutant showed loss of the continuity between aortic valve and mitral valve, with a gap between the mitral and aortic valves (**m**, yellow arrowhead). Instead of aorta being to the right and posterior to the pulmonary artery (**g**), the *Lrp1*^*m/m*^ mutant exhibited a side-by-side and rightward aorta (AO) to pulmonary artery (PA) relationship (**n**). Scale bars: 0.5 mm.

Mice harboring the *Lrp1*^*C4232R*^ mutation exhibit homozygous lethality at E15.5, with rare stillborn pups. In addition to CHD, *Lrp1*^*m/m*^ embryos also exhibited extracardiac defects such as micrognathia and cleft palate, with some mutants exhibiting gastroschisis with liver protruding outside the abdominal cavity (Supplementary Fig. 1). Cardiovascular assessments conducted using fetal echocardiography^8^ and episcopic confocal microscopy (ECM)^9^ showed outflow tract (OFT) malalignment defects in the *Lrp1*^*m/m*^ mutants. Side-by-side positioning of the OFT was typically observed with blood flow across the ventricular septum, indicating possible double outlet right ventricle (DORV) (Fig. 1i, l, n). Also, commonly seen was atrioventricular septal defect (AVSD) with blood flow mixing between all four cardiac chambers (Fig. 1i–k).

During heart development, a single outflow forms that subsequently divides to generate the aortic and pulmonary arteries. Initially, both arteries are connected to the RV, but with development, the aortic root shifts, becoming wedged between the AV valves, causing the aorta to lie over the left ventricle^10^. During this same developmental window, endocardial/epithelial–mesenchymal transition (EMT) occurs^11^, generating cardiac cushions that later remodel to form the semilunar and AV valves^12^. ECM imaging showed all the mutants have CHD, the majority (90.63%) exhibiting DORV with AVSD (Supplementary Fig 2), 6.25% having isolated DORV, and 3.12% with isolated AVSD (Fig. 1, Supplementary Fig. 2).

### Lineage-specific roles of *Lrp1* in cardiac development

**Fig. 2:**
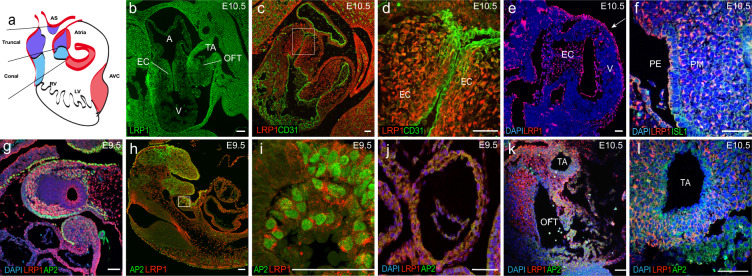
LRP1 expression in different cell linages that are important for cardiac development in the developing heart. Panel **a** is schematic representation of embryonic heart showing AVC and OFT (conal part and truncal part). Immunostaining in E9.5 and E10.5 embryos demonstrated LRP1 is expressed in mesenchymal cells in the endocardial cushion, with no expression seen in the CD31 positive endocardium (**b**–**d**) at E10.5; epicardium (e white arrow) at E10.5 pharyngeal endoderm and pharyngeal arch (**b**, **f**) at E10.5, neural crest/mesenchymal cells (**g**–**i**) at E9.5; outflow tract (**i**) at E9.5, outflow tract (**j**) at E10.5 and truncal artery (**k**) at E10.5. LRP1 and Islet-1 (ISL1) are expressed in (**f**) pharyngeal endoderm and pharyngeal mesoderm. LRP1 and AP2α both expressed in the neural crest/mesenchymal cells (**g**–**i**) and outflow tract (**j**–**l**). **i** The magnified view from the white box of figure (**h**). Scale bars: 50 µm.

To determine whether *Lrp1* could play a role that is autonomous to cell populations essential for development of the OFT and endocardial cushions, we examined LRP1 expression in the E10.5 embryo using immunohistochemistry with a LRP1 antibody. LRP1 expression was observed in many cell types in the heart, including the OFT (Fig. 2b, j–l), the mesenchymal cells in the atrioventricular (AV) canal (Fig. 2b–e), the OFT cushion (Fig. 2b, k) and in the epicardium (Fig. 2e). Antibody staining with ISL1, a second heart field (SHF) marker^13^, showed colocalization of LRP1 and ISL1 in pharyngeal mesoderm (PM) and endoderm (PE) (Fig. 2f). AP2α, a neural crest marker^14^, also colocalized with LRP1 in the OFT (Fig. 2j–l) and CNC/mesenchymal cells (Fig. 2g–i). Surprisingly, LRP1 expression was not observed in the endocardium/endothelial cells (Fig. 2b, c). The endocardium normally undergoes EMT to generate mesenchymal cells in the cardiac cushions^11^. These AV cushions function as primitive valves that later will remodel into mature cardiac valves.

Since *Lrp1* expresses in different cell lineages in the critical period in the developing heart, we used a floxed *Lrp1 (Lrp1*^*f/f*^*)* allele^15^ with different Cre drivers to orchestrate conditional *Lrp1* deletion in cell lineages associated with cardiac development. The Cre drivers used included Wnt1^16^, Mef2c-anterior heart field-Cre targeting cells^17^, Nkx2–5^18^, Tie2^19^, Twist2^20,21^, and Nfat1c^22^, in the CNC (Wnt1-Cre^16^), anterior heart field/PM (Mef2c-Cre^17^), first heart field (Nkx2–5-Cre^18^), PE/PM (NKx2–5-Cre^18^), endothelial/endocardial cells and mesenchymal cells of AV cushion (Tie2-Cre^19^), mesenchymal cells of AV cushion (Twist2-Cre^20,21^), and endothelial/endocardial cells (Nfat1c-Cre^22^), respectively.

**Fig. 3:**
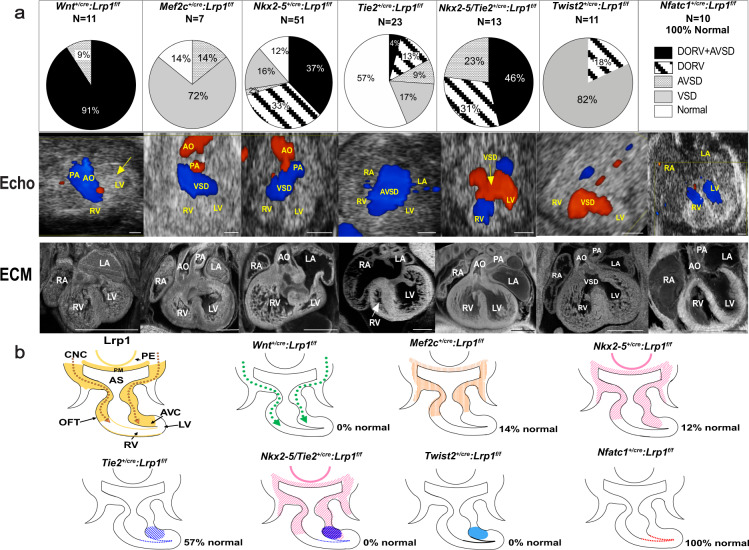
Conditional Cre deletion of floxed *Lrp1* allele. *Lrp1* conditional knockout in different cell lineages during cardiac development. **a** Pie charts illustrate the percentage of each cardiac phenotype. Representative echocardiograms and ECM pictures in each conditional knockout are demonstrated in (**a**). Targeting *Lrp1* deletion in cardiac neural crest cells using *Wnt1-Cre* recapitulated the cardiac phenotype of *Lrp1*^*m/m*^ mutants with AVSD and DORV in 91% of *Wnt1*^*+/Cre*^: *Lrp1*^*f/f*^ mutants, yellow arrow indicated pericardial hemorrhage. Deletion of *Lrp1* in the AHF derivatives using the *Mef2c-AHF-Cre* yielded septation defects. *Nkx2–5-*Cre mediated deletion of *Lrp1* yielded mostly DORV with or without AVSD. Totally, 43% of mutants generated with the *Tie2-Cre* drivers had septation defects. Combined deletion of *Lrp1* mediated by both *Tie2-Cre* and *Nkx2–5-Cre* recapitulated the high penetrance of the DORV/AVSD phenotype observed in the *Lrp1*^*m/m*^ mutant and increase the penetrance of AVSD. Deletion of *Lrp1* in the mesenchymal cells of AV cushion with Twist2-Cre drivers showed 82% of mutants with membranous VSD and 18% mutants with DORV. There were no cardiac abnormalities with the *Nfatc1-Cre* drivers, consistent with absence of *Lrp1* expression in the endocardium of atrioventricular cushion. **b** Schematic representation of LRP1 expression (yellow) in developing heart around E10.5–E11.5. *Lrp1* expresses in pharyngeal endoderm (PE) and pharyngeal mesoderm (PM), mesenchymal cells of outflow tract cushion, atrioventricular cushion, neural crest cell migration pathway as well as epicardial cells. Ablation of *Lrp1* in neural crest cells using *Wnt1-Cre* (*Wnt1*^*+/Cre*^:*Lrp1*^*f/f*^) is illustrated in dotted green arrows. Ablation of *Lrp1* using *Mef2c-AHF-Cre* (*Mef2c-AHF*^*+/cre*^: *Lrp1*^*f/f*^) is illustrated in orange. Ablation of LRP1 expression using *Nkx2–5-Cre* (Nkx2–5^*+/Cre*^:*Lrp1*^*f/f*^) is illustrated in pink, specifically in PM, PE, AHF, OFT cushion and AV canal cushion. Ablation of *Lrp1* expression using *Tie2-Cre* (*Tie2*^*+/Cre*^: *Lrp1*^*f/f*^) is illustrated in navy-blue hatch (mesenchymal cells of atrioventricular cushion) and navy-blue dots (endothelium of atrioventricular canal and endocardium of ventricle). The ablation of *Lrp1* in double knockout of *Nkx2–5-Cre* and *Tie2-Cre* (Nkx2–5^*+/Cre*^ *Tie2*^*+/Cre*^: *Lrp1*^*f/f*^) is illustrated in purple and navy blue. Ablation of *Lrp1* in *Twist2-Cre* is expressed in azure (royal blue). Ablation of *Lrp1* expression in *Nfatc1-Cre* is expressed in red. Scale bars: 0.5 mm.

*Wnt1-Cre*^16^: To examine *Lrp1* requirement in the CNC lineage, we conditionally ablated *Lrp1* using the neural crest-specific Cre recombinase *Wnt1-Cre*^16^. *Wnt1*^*+/cre*^: *Lrp1*^*f/f*^ mutant embryos did not survive past E14.5. Significantly, 100% of the *Wnt1*^*+/cre*^: *Lrp1*^*f/f*^ embryos exhibited CHD: 91% with DORV and AVSD, and 9% with AVSD. This replicates the CHD phenotype and with same penetrance as seen in the *Lrp1*^*m/m*^ mutant (Fig. 3). These findings suggest CHD in the *Lrp1*^*m/m*^ mutant may arise from a cell autonomous requirement for *Lrp1* in the CNC cells.

We next used *Mef2c-AHF-Cre*^17^ to test whether *Lrp1* in the anterior heart field is essential for formation of the OFT^17^. With *Mef2c-AHF-Cre*^17^ deletion, 14% of the *Mef2c-AHF*^*+/Cre*^: *Lrp1*^*f/f*^ embryos exhibited no CHD, while 72% had a membranous VSD, with one mutant (14%) exhibiting a partial AVSD (Fig. 3). None of *Mef2c-AHF* ^*+/Cre*^*:Lrp1*^*f/f*^ exhibited either DORV or DORV/AVSD as seen in the original *Lrp1*^*m/m*^ mutant.

*Nkx2–5* driven Cre^18^: Using the *Nkx2–5-*Cre driver^18^, we investigated the requirement for *Lrp1* in myocardial cells from the first and SHF^18^. Analysis of 51 *Nkx2–5*^*+/cre*^:*Lrp1*^*f/f*^ embryos showed 88% have CHD. This included 37% (*N* = 19) with AVSD and DORV, 33% (*N* = 17) with isolated DORV, 16% (*N* = 6) with membranous VSD, and 2% (*N* = 1) with unbalanced AVSD with dominant left ventricle (Fig. 3).

Tie2^19^, Twist2^20,21^, and Nfatc1^22^ driven Cre: Using *Tie2-Cre*^19^, we targeted *Lrp1* deletion in the endothelial/endocardial lineage, and also cells in the cushion mesenchyme^19^. With *Tie2-Cre*^19^ deletion, only 43% of the 23 Cre deleted embryos (*Tie2*^*+/cre*^: *Lrp1*^*f/f*^) have cardiac defects. This included 17% with membranous VSD (*N* = 4), 13% (*N* = 3) with DORV/subaortic VSD, 9% (*N* = 2) with isolated AVSD, and one (4%) with DORV/AVSD (Fig. 3). As *Lrp1* is not expressed in endothelial/endocardial cells but is highly expressed in the cushion mesenchyme, this suggested that the CHD resulting from *Tie2-Cre*^19^ deletion may reflect a requirement for *Lrp1* function in endocardium derived cushion mesenchyme. Hence, we further assessed *Lrp1* deletion using the mesenchymal specific *Twist2-Cre*^20,21^ and endocardial specific *Nfatc1*-Cre^22^. All the mutant embryos with *Twist2-Cre*^20,21^ deletion (*Twist2*^*+/cre*^*: Lrp1*^*f/f*^) had septal defects with membranous VSD in 82% (*N* = 9) and isolated DORV in 18 (*N* = 2). In contrast, all ten embryos generated with *Nfatc1-Cre*^22^ deletion (Nfatc1^*+/cre*^: *Lrp1*^*f/f*^) had normal cardiovascular anatomy, suggesting the cushion mesenchymal cells targeted by *Tie2-Cre* are not of endocardial origin (Fig. 3).

To test whether *Lrp1* in myocardial cells and in non-endocardium derived cushion mesenchyme contribute synergistically to heart development, we knocked out *Lrp1* using the *Nkx2–5-Cre*^18^ and *Tie2-Cre9*^19^ drivers simultaneously. This resulted in synergistic effects, with 100% of the embryos exhibiting CHD, as compared to 43% with *Tie2*^*+/Cre*^:*Lrp1*^*f/f*^ and 88% with *Nkx2–5* ^*+/Cre*^*:Lrp1*^*f/f*^ (Fig. 3). There was a tenfold increase in the incidence of DORV with AVSD—46% with *Tie2*^*+/Cre*^*/Nkx2–5*^*+/Cre*^:*Lrp1*^*f/f*^ double Cre deletion vs. 4% with *Tie2*^*+/Cre*^:*Lrp1*^*f/f*^. Overall, there was a marked increase in AVSD—69% with the *Tie2*^*+/Cre*^*/Nkx2–5*^*+/Cre*^:*Lrp1*^*f/f*^ double Cre deletion vs. 13% with *Tie2* ^*+/Cre*^: *Lrp1*^*f/f*^ and 39% with *Nkx2–5*^*+/Cre*^*:Lrp1*^*f/f*^ (Fig. 3). These results suggest both myocardial cells and non-endocardium derived cushion mesenchyme are essential for supporting *Lrp1*-mediated heart development.

Together, these data show *Lrp1* function is primarily required in the CNC lineage, with a secondary requirement in the myocardial cells of the first and SHF and in cushion mesenchyme of non-endocardial origin.

### OFT and defects in development of the endocardial cushion

**Fig. 4:**
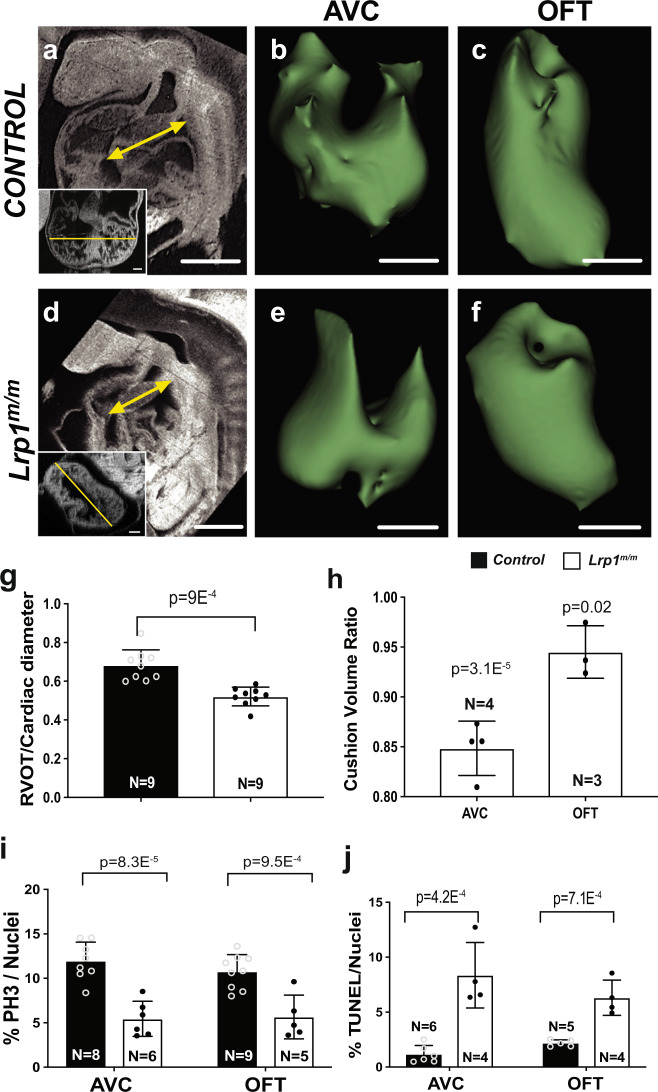
*Lrp1*^*m/m*^ mutant hearts exhibit decreased OFT length, decreased AVC and OFT cushion volume, decreased proliferation and increased apoptosis at E10.5. ECM in the sagittal plane of an E10.5 wildtype (**a**) and *Lrp1*^*m/m*^ mutant (**d**) embryos are shown, indicating reduction in length of mutant OFT versus control (yellow arrow in (**a**, **d**)). Insert panels in **a** and **d** demonstrated the cardiac diameter (yellow line) in the corresponding embryo. **g** Quantitative measurement using histopathology images showed a significant decrease in the length of the OFT with cardiac diameter normalization in the homozygous *Lrp1*^*m/m*^ mutant hearts as compared to the controls (combined wildtype and *Lrp1*^*+/m*^ hearts). Three-dimensional (3D) reconstructions of AVC (**b**, **e**) and OFT (**c**, **f**) were generated from ECM of *Lrp1*^*m/m*^ mutant hearts and controls at E10.5. **h** Quantitative measurement using histopathology images and 3D slicer to process the images showed a significant decrease in the volume (with normalization of cardiac diameter) of the AVC and OFT in the homozygous *Lrp1*^*m/m*^ mutant hearts as compared with the controls (combined wildtype and *Lrp1*^*+/m*^ hearts). **i** Quantitative results of cell proliferation using the ratio of positive pH3 cells/total cushion cells in AVC and OFT cushion. **k** Quantitative results of the ratio of positive TUNEL cells/total cushion cells. Statistical comparison was performed using unpaired two-way Student′s *t* test. Error bars show standard deviation. Scale bars: 0.5 mm in (**a**, **d**); 0.1 mm in (**b**, **c**, **e**, **f**).

To determine the developmental processes contributing to the OFT malalignment and endocardial cushion defects in the *Lrp1*^*m/m*^ mutants, we conducted ECM imaging and 3D reconstructions of the E10.5 heart from *Lrp1*^*m/m*^ mutant and littermate control embryos. Measurements of the OFT length with cardiac diameter normalization showed a significant reduction in OFT length of the *Lrp1* mutant embryos compared to littermate controls (Fig. 4a, d, g). Volumetric analysis of the atrioventricular (AV) and outflow (OFT) cardiac cushions with cardiac diameter normalization showed a significant reduction in the volume of both cardiac cushions in the *Lrp1*^*m/m*^ mutant embryos (Fig. 4b, c, e–h). Analysis of cell proliferation with pH3 staining^23^ revealed a decrease in mitotic cells in the AVC/OFT cushion of *Lrp1*^*m/m*^ embryos (Fig. 4i, Supplementary Fig 3a). Transferase-mediated dUTP-biotin nick-end labeling (TUNEL) staining^24^ showed increased apoptosis in the endocardial cushions of *Lrp1*^*m/m*^ mutants (Fig. 4j, Supplementary Fig. 3b).

**Fig. 5:**
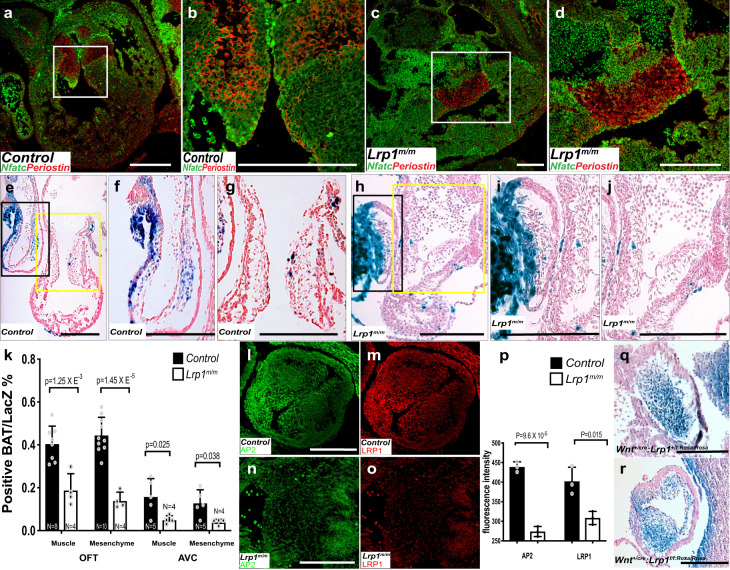
Abnormal atrioventricular and outflow tract cushion development was observed in *Lrp1*^*m/m*^ mutants. Immunostaining of NFATC-1 (green) and Periostin (red) in wildtype (**a**, **b**) and *Lrp1*^*m/m*^ mutant (**c**, **d**) embryos at E12.5. **b**, **d** Magnified views of the white box from (**a**, **c**). Compared with the well-formed two atrioventricular valves in the wildtype control, *Lrp1*^*m/m*^ mutant demonstrated primitive undivided endocardial cushion morphology. Representative images from X-gal and Eosin stained tissue sections from E10.5 control (*Lrp1*^*m/+*^) (**e**–**g**) and *Lrp1*^*m/m*^ mutant (**h**–**j**). **f**, **i** The high magnified view of the black box **e** and **h** (OFT). **g**, **j** The high magnified view of the yellow box from (**e**, **h**) (AV cushion). X-gal staining **e**–**j** demonstrated diminished expression of LacZ expression in the *Lrp1*^*m/m*^ AVC (**e**, **f**, **h**, **i**) and OFT cushion (**e**, **g**, **h**, **j**). **k** Quantitative analysis of the percentage of positive BAT/LacZ cells in the muscle and mesenchyme portion of OFT and AVC. *Lrp1*^*m/m*^ mutant had hypocellular AVC and OFT cushions (**h–j**). **l**–**o** Immunostaining of AP2α (green) and LRP1 (red) in *Lrp1*^*m/m*^ mutant (**n**, **o**) and control (**l**, **m**). *Lrp1*^*m/m*^ mutant had decreased expression of AP2α and LRP1 in the outflow tract at E10.5–E11.5 (**l**–**o**). **p** Quantitative analysis of the fluorescence intensity of AP2α and LRP1 demonstrated decreased expression of both AP2α and LRP1 in *Lrp1*^*m/m*^ mutant OFT. (**q, r**) LacZ staining of OFT in *Wnt1*^*+/cre*^*: Lrp1*^*f/f/rosa/rosa*^ and control (*Wnt1*^*+/cre*^*: Lrp1*^*f/+/rosa/rosa*^), which demonstrated decreased LacZ expression in the *Wnt1*^*+/cre*^*: Lrp1*^*f/f/rosa/rosa*^ mutant. Scale bars: 200 μm. Statistical comparison was performed using unpaired two-way Student's *t* test. Error bars show standard deviation.

Further analysis of the E12.5 *Lrp1*^*m/m*^ mutant heart revealed a single primitive, undivided AV cushion as compared to two distinct, well separated AV cushions (the mitral and tricuspid valve anlage) in wild-type embryos (Fig. 5a–d). Immunostaining of Periostin, an extracellular matrix protein expressed in the cardiac cushions, revealed deformed and immature endocardial cushions in the *Lrp1*^*m/m*^ mutants (Fig. 5a–d).

Given the known importance of Wnt signaling in cardiac valvular morphogenesis, we crossed a Wnt BAT-LacZ reporter^25,26^ into the *Lrp1*^*m/m*^ mutant line to assess in situ Wnt signaling in the developing cardiac cushions. X-gal staining revealed a marked decrease in LacZ expressing cells in the AV and OFT cushions of the E10.5 *Lrp1*^*m/m*^ mutant embryos as compared to control (Fig. 5e–k). This was associated with hypocellularity characterized by decreased abundance of mesenchymal cells in the endocardial and OFT cushions (Fig. 5e–j), these results consistent with the reduced cell proliferation and increased apoptosis observed earlier in the E10.5 embryo AVC and OFT cushions (Fig. 4i, j).

To further delineate the role of LRP1 in OFT development, immunostaining of AP2-α^14^, a marker for neural crest cell and LRP1 in E10.5 embryos was used. Compared with controls (Fig. 5l, m), *Lrp1*^*m/m*^ mutants demonstrated significant decreased expression of AP2-α and LRP1 (Fig. 5n–p) in the developing OFT. Consistent with the conditional deletion of LRP1 in the neural crest lineage^16^ results in OFT defects (DORV with AVSD, DORV), a Cre lineage tracing of the neural crest cells, *Wnt1-Cre*^16^ with Rosa/LacZ reporter^27^, demonstrated decreased expression of the Wnt1/LacZ cells in the OFT at E10.5 embryos (Fig. 5q, r), which is consistent with the decrease in Wnt BAT-LacZ expression in the AV and OFT cushions of *Lrp1*^*m/m*^ mutants (Fig. 5e–k). These data indicate neural crest cells require LRP1 to migrate to the cardiac OFT properly.

### Gene expression analysis indicates EMT and cell migration defects

**Fig. 6:**
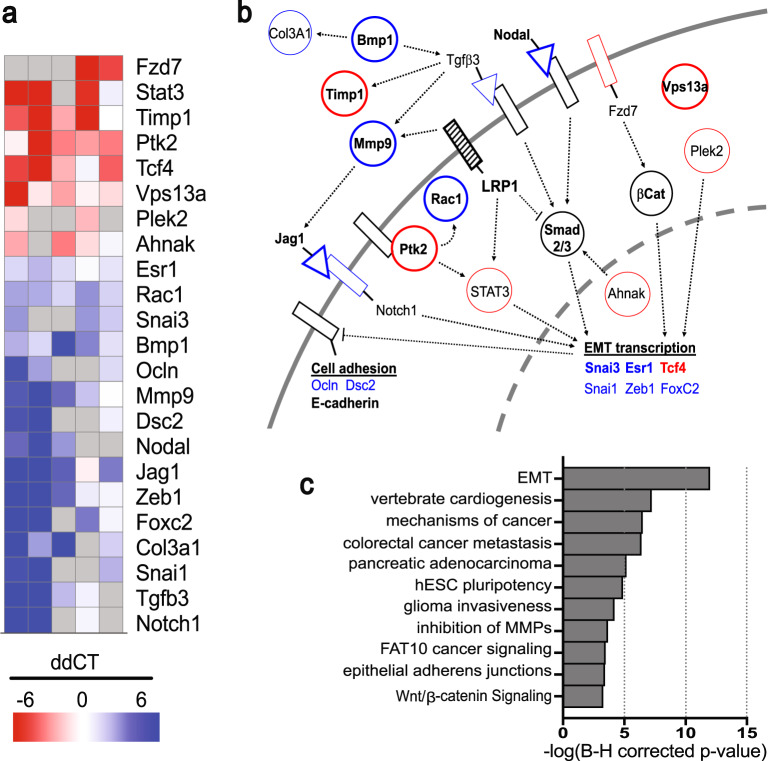
Quantitative differential expression of EMT-related genes by real-time PCR. Quantitative comparison of EMT-related gene expression in *Lrp1*^*m/m*^ vs. WT in the endocardial cushion at E10.5, prepared using Morpheus (Broad Institute) (**a**). **a** Genes which were repeatably over- (red) or under-expressed (blue) are shown. After discarding measurements with unacceptably low copy number (gray; raw Ct > 40), replicate ddCt values were filtered with an outlier test (ROUT, *Q* = 10%) and samples with three replicates were tested against a H0 that ddCt = 0 using a one-sample two-tailed *t* test. Genes that passed (*p* < 0.05) are shown, along with other strongly responsive genes (*p* ≥ 0.05, but mean > variance). Red indicates genes upregulated in *Lrp1*^*m/m*^ and blue indicates downregulated. The functional relationships between these genes is shown in (**b**); bolded names or outlines indicate genes with significant overexpression or underexpression and lighter names or outlines indicates strongly responsive but not significant. Color coding is the same as in (**a**); black outlines or names represent key genes that are not overexpressed or underexpressed in this analysis. The major cellular pathways represented by this gene set are shown in (**c**), as determined by Ingenuity Pathway Analysis.

Since the hypocellularity associated with both the *Lrp1*^*m/m*^ endocardial and OFT cushions suggest a hindrance in the ability of *Lrp1*^*m/m*^ cells to undergo proper EMT, leading to cardiac cushion defects, we next sought to investigate the molecular perturbation underlying the cardiac cushion defects in the *Lrp1*^*m/m*^ mutants. We used a polymerase chain reaction (PCR) array focused on EMT to examine gene expression alterations in the E10.5 AV cushions (Fig. 6a, b). We conducted these real-time PCR experiments on five independent mutant and littermate control embryo pairs. When we analyzed altered genes using Integrated Pathway Analysis, several impacted pathways related to EMT stood out (Fig. 6c). These included cell–cell adhesion and cell invasiveness, vertebrate cardiogenesis, cancer metastasis, glioma invasiveness, inhibition of matrix metalloproteinase, and epithelial adherens junctions (Fig. 6b, c). Also observed were changes in Wnt/catenin signaling^26,28^ and gene expression changes related to bone morphogenetic protein (Bmp)/transforming growth factor-β (Tgf-β)^12^, and Notch signaling^12^—all cell signaling pathways with important roles in valvular morphogenesis^12^ (Fig. 6b).

### Cell migration defects due to reduced FA turnover

**Fig. 7:**
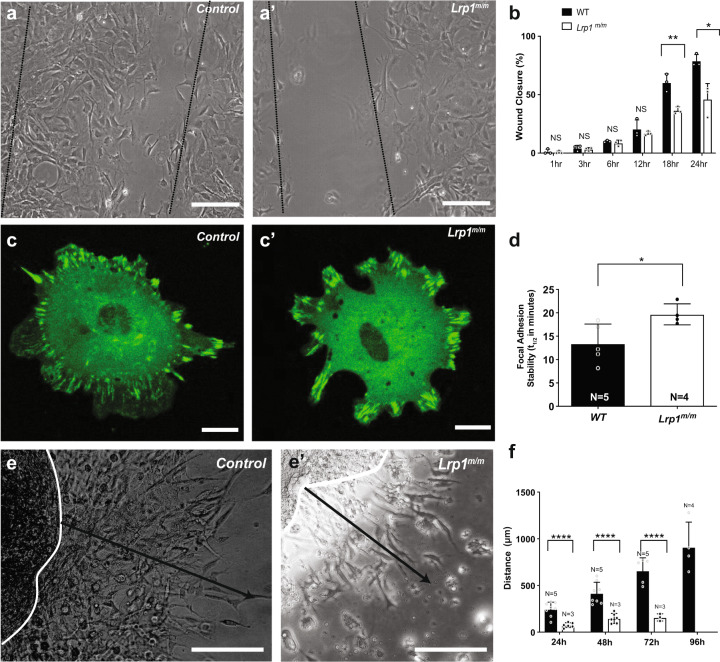
*Lrp1* mutation causes migration defects. **a**, a′ Representative image of wound scratch assay from (**a**) a wild-type control and (a′) a mutant MEF, 18 h after scratch. **b** Quantitation of wound gap closure showed *Lrp1*^*m/m*^ mutant MEFs have decreased cell motility compared to control with decreased filling of the scratch “gap” compared with controls. % indicates the percentage of closing the gap, 100% means close the gap completely. **c** Wild-type and (c′) *Lrp1*^*m/m*^ mutant fibroblast cells were transfected with vinculin-GFP and vinculin turnover was measured using live-cell imaging. **d** Quantitative measurement revealed slower focal adhesion turn over in MEF from *Lrp1*^*m/m*^ mutant, as measured by the half-life of the overlap between segmented focal adhesions at time 0 and time *n*. **e**, e′ Representative imagine of AVC explant migration assay from a wild-type control (**e**) and *Lrp1*^*m/m*^ mutant (e′). Black line indicated the migration distance at 48 h. **f** Quantitative analysis of the distance of migration of endocardial cushion explants from E10.5 control and *Lrp1*^*m/m*^ embryos showed near complete loss of cell migration from the *Lrp1*^*m/m*^ mutant explants Statistical comparison was performed using unpaired two-way Student′s *t* test. Error bars show standard deviation.

Given the importance of directional cell migration in the targeting of NCC to the heart, we tested whether the *Lrp1*^*C4232R*^ mutation inhibits the migration of fibroblasts from *Lrp1*^*m/m*^ embryos. This entailed growing the MEFs to confluency, then making a scratch in the monolayer to create a wound gap to stimulate cell migration. *Lrp1*^*m/m*^ mutant MEFs showed a significant reduction in the rate of wound closure (Fig. 7a, a′, b). This was due to alterations in the speed of cell locomotion, but no change in the directionality of cell migration was observed (Fig. 7a, a′). Consistent with this, Golgi apparatus oriented normally with the direction of cell migration, indicating the ability of cells to polarize was unaffected (Supplementary Fig. 4a, d).

Notably, LRP1 regulates the trafficking of integrins, which regulate focal adhesions (FA) and link the extracellular matrix to the actin cytoskeleton for cell migration and matrix adhesion^29^. To determine whether our *Lrp1*^*m/m*^ mutation alters the structure or behavior of FA, we used quantitative confocal imaging analysis with an antibody to vinculin to measure the number, area, and length of FA in *Lrp1*^*m/m*^ MEFs. This analysis showed no net change in focal contacts in mutant vs. wild-type MEFs (Supplementary Fig. 4b, c, e–g). Since FA are dynamic structures regulated by turnover, we conducted live cell imaging with transient transfection of a vinculin-GFP reporter to measure the FA turnover rate. Our analysis showed turnover in the FA “footprint” (Fig. 7c, c′, d, Supplementary Video 1) is significantly reduced in *Lrp1*^*m/m*^ MEFs, consistent with the reduced cell migration rate seen for the mutant MEFs in the wound closure assay (Fig. 7a, a′, b). As formation of the endocardial cushion involves EMT, a process associated with cell invasion and migration^11^, we tested migration of cushion cells by plating AV cushion explants on a collagen gel matrix. This analysis showed a marked reduction in migration (Fig. 7e, e′, f) of *Lrp1*^*m/m*^ mutant explants.

### LRP1 C4232R mutation alters endocytic trafficking

**Fig. 8:**
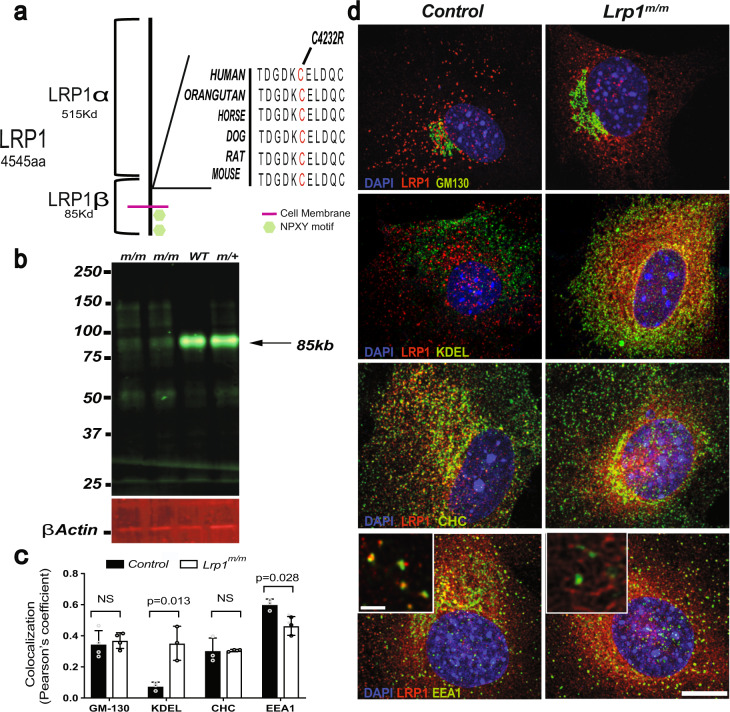
LRP1 C4232R mutant protein affects the expression of the 85 kD (LRP1-β) domain and has increased retention in the endoplasmic reticulum. Schematic diagram of LRP1 protein. **a** LRP1 consists of 4545 amino acids. The mutant line 1554 (MGI 96828) harbors a missense (C4232R) mutation in the extracellular region encoding the EGF repeat domain of LRP1. The cysteine residue mutated at position 4232 is conserved among species. **b** Immunoblotting analysis showed strongly reduced expression of the 85 kD LRP1 protein in liver lysates from the *Lrp1*^*m/m*^ homozygous mutant, as compared with *Lrp1*^*+/m*^ and wild-type embryos. **c** Quantitative measurement of colocalization between LRP1 and the markers of cellular compartments using Pearson′s coefficient demonstrates (**c**, **d**) *Lrp1*^*m/m*^ has increased localization to the ER (KDEL) and decreased localization in early endosomes (EEA1). Error bars showed mean standard deviation. **d** Immunostaining for LRP1 and markers for the Golgi apparatus (GM130), endoplasmic reticulum (ER) marker (KDEL), clathrin marker (CHC), and the early endosome marker (EEA1). Scale bars: 10 µm in (**d**); 2 µm in magnified box in (**d**).

Since the *Lrp1* missense mutation C4232R is situated in a highly conserved cysteine residue within one of the extracellular EGF repeat domains of the protein that bind membrane proteins/ligands^5,6^, we next sought to determine how *Lrp1*^*C4232R*^ is altered at the protein level (Fig. 8a). Western blot of protein extraction from the liver of *Lrp1*^*m/m*^ showed the LRP1 protein is expressed, though at reduced levels, suggesting that the protein could be degraded during or after synthesis (Fig. 8b).

To test whether trafficking of LRP1^C4232R^ is normal, we used confocal microscopy to measure its colocalization with various cell compartments, including an endoplasmic reticulum (ER) resident protein (KDEL), a Golgi marker (GM130), clathrin heavy chain (CHC) (Fig. 8c, d), and the early endosome marker (EEA1)^30^ (Fig. 8c, d). In wild-type MEFs, LRP1 showed the expected strong colocalization with early endosome marker EEA1, but less so with KDEL, GM130, and CHC (Fig. 8d). In contrast, in *Lrp1*^*m/m*^ MEFs, LRP1 had reduced co-localization with early endosomes, but strong co-localization with KDEL (Fig. 8c, d), indicating ER retention of the mutant LRP1^C4232R^ protein. These results suggest the substantial reduction in the 85 kD LRP1 β-chain seen in the Western blot is likely due to retention and ER-associated degradation of misfolded LRP1 protein or due to problems cleaving the 600 kD protein to 515 and 85 kD subunits.

## Discussion

We showed that a missense mutation in *Lrp1*, *Lrp1*^*C4232R*^, causes prenatal lethality with DORV and AVSD. This was embryologically associated with shortening of the OFT and hypoplastic/under-development AV cushions. The *Lrp1*^*C4232R*^ mutation is hypomoprhic, with retention in the ER and marked reduction of the 85 kD LRP1 β-chain, likely a reflection of degradation of misfolded protein or problems during cleavage of the of 600 kD LRP1 protein. Using Cre drivers we found the CHD phenotypes and disease penetrance of the *Lrp1* mutation can be fully replicated with *Wnt1-cre*, suggesting that LRP1 function during OFT development is required cell autonomously in the CNC cells.

While *Lrp1* deletion mediated by other Cre drivers also generated cardiac defects, unlike *Wnt-1*^*+/Cre*^:*Lrp1*^*f/f*^ in which 100% of the mutant embryos exhibited CHD, fewer cardiac defects were seen in *Nkx2–5*^*+/Cre*^*:Lrp1*^*f/f*^, *Mef2c-AHF*^*+/Cre*^*:Lrp1*^*f/f*^ and *Tie2*^*+/Cre*^*:Lrp1*^*f/f*^ deletion embryos (12%, 14%, and 57% had normal cardiac anatomy, respectively). These Cre drivers have broad expression domains encompassing embryonic tissues with *Lrp1* expression found along the CNC migratory path, including myocardium of the first/SHF, PE and PM, and cushion mesenchyme. Hence, *Lrp1* expression in these additional cell lineages may provide signaling function that can ensure quantitative recruitment of neural crest cells to the heart. Consistent with this model, *Nkx2–5*^*+/Cre*^*/Tie2*^*+/Cre*^: *Lrp1*^*f/f*^ double Cre deletion exert additive effects, yielding more severe phenotypes with higher heart defect penetrance than either *Nkx2–5*^*+/Cre*^ or *Tie2*^*+/Cre*^ Cre driver alone.

The requirement for CNC cells in heart development is well described^31–34^, but what function they provide remains largely unknown. Neural crest cells are required for OFT alignment and septation^28,31–34^, and in the development of the endocardial cushions^26^. Proper great artery connections with their respective ventricles involve rotation of the developing great arteries and wedging of the aorta between the AV valves to achieve mitral-aortic fibrous continuity^10^. Our observations in the *Lrp1*^*m/m*^ mutants with decreased expression of AP2-α expression and decreased Wnt BAT-LacZ expression in the developing OFT would suggest that CNC cells may impact this process through regulating OFT lengthening by providing migratory cues for recruiting SHF cells and endothelial-derived cells to the distal OFT. Conditional deletion of CNC using Wnt1-Cre driver had decreased expression of neural crest cells in the OFT cushion at E10.5 indicating the necessity of LRP1 expression in the developing heart for proper CNC cell migration. Consistent with this, CNC ablation can lead to shortening of the OFT with fewer myocardial cells added to the distal OFT^31^.

Our analysis of the cardiac cushion explants and MEFs from the *Lrp1*^*m/m*^ mutant embryos revealed a cell migration defect. This was associated with reduction in the rate of FA turnover. LRP1 is known to regulate cell surface recycling of integrins that link the actin cytoskeleton with the extracellular matrix in FA^29^. Our *Lrp1*^*C4232R*^ mutation is situated in the β-chain harboring the endocytic NPxY1/NpXy2 and YXXL motifs. Previous studies of MEFs with a *Lrp1* knock-in replacement of the NPxY2 motif showed elevated cell surface β1-integrin expression^29^. This was associated with increased cell adhesion and decreased cell migration^29^, results similar to our findings. Based on these findings, we propose a primary CNC migration defect may drive the OFT malalignment phenotype in the *Lrp1*^*m/m*^ mutant. We note a previous study of mice with *Nkx2–5-Cre* mediated KO of *Fak* encoding FA kinase yielded CHD comprising DORV or overriding aorta^35^. As *Nkx2–5-Cre* deletion of *Lrp1* also generated DORV, but with reduced penetrance compared to the *Wnt1*^*+/Cre*^: *Lrp1*^*f/f*^, this would suggest there is a secondary requirement for *Lrp1* in cells of the SHF to orchestrate normal OFT lengthening.

Gene expression analysis of the cardiac cushions showed disruption of Wnt/catenin signaling, as well as perturbation of Bmp/Tgfβ, and Notch signaling. KO mice with disruption of these three cells signaling pathways all show OFT defects, including DORV^36^. In contrast, OFT malalignment defects are not observed with disruption of NFAT, a signaling pathway also known to play important roles in valvular morphogenesis^37,38^. The disruption of these cell signaling pathways may secondarily contribute to the cushion mesenchyme hypoplasia. This cushion defect may also cause AVSD and perturb wedging of the aorta to disrupt OFT alignment.

Unexpectedly, we found evidence for altered EMT in *Lrp1*^*m/m*^ mice. Cardiac cushions in the e10.5 OFT, which depend strongly on EMT for development^38^, are dysmorphic and hypocellular in *Lrp1*^*m/m*^ mice. Fibroblasts derived from *Lrp1*^*m/m*^ embryos had reduced FA turnover and reduced directional migration both in 2D assays and in 3D explant cultures. Importantly, this defect was not caused by loss of directional migration, as the Golgi apparatus oriented normally towards a gap ‘scratched′ in a confluent monolayer. These data suggest that the EMT pathway could be a key effector of Lrp1 during heart development, a question that we intend to pursue more detail in subsequent studies.

In summary, we show, to our knowledge, for the first time, deficiency in an endocytic trafficking protein in the cardiac NCC can cause CHD from disruption of OFT lengthening and the expansion of AV cushion mesenchyme. Further investigations into how disruption of endocytic trafficking may affect neural crest function may help elucidate the developmental etiology for DORV and give insights into the broader role of endocytic trafficking in CHD pathogenesis.

## Methods

### Recovery of the *Lrp1* mutation and mouse breeding

Mouse studies were conducted under an approved University of Pittsburgh Institutional Animal Care and Use of Committee protocol. Breeding for the mutagenesis screen was described previously^4^, which used both male and female mice between 6 weeks and 6 months of age in a C57BL6/J background. Genomic DNA for line 1554 (MGI: 5437079) was harvested from a mutant embryo displaying AVSD, DORV, and pulmonary stenosis, then sequence captured using Agilent SureSelect Mouse All Exon Kit V1 and sequenced using Illumina HiSeq 2000 with a goal of 50× as the minimum target-sequence coverage (BGI America). Sequences were aligned to the C57BL/6J mouse reference genome (mm9) then analyzed using CLCBio Genomic Workbench and the Genome Analysis Toolkit (GATK) software. Annotation of sequence variants was performed using ANNOVAR (http://www.openbioinformatics.org/annovar/) then filtered against custom in-house scripts as well as dbSNP128 (https://www.ncbi.nlm.nih.gov/projects/SNP/snp_summary.cgi?build_id=128). Ten homozygous coding mutations were identified (Supplementary Table 1) More than 10 generations of breeding line 1554 has further validated the *Lrp1* mutation as responsible for the displayed phenotypes.

### Mouse lines

Line 1554 (MGI:5437079) harboring a *Lrp1*^*C4232R*^ missense mutation (*Lrp1*^*m/m*^) previously recovered from a large scale mouse mutagenesis screen^4^ is maintained in a C57BL6/J strain background, having been backcrossed for more than ten generations with C57BL6/J mice. A *Lrp1*^*flox/flox*^ (*Lrp1*^*f/f*^) allele was obtained from the Jackson Laboratory [*Lrp1*^*tm2Her*^*/J* #012604]^15^ which was generated by inserting a floxed Neo cassette and a single *loxP* site into the downstream of exon 2 of *Lrp1* gene. This floxed allele (*Lrp1*^*f/f*^) was crossed with mice carrying different Cre drivers for targeting Lrp1 deletion in different cell lineages: this includes intercrossing with *Wnt1-Cre* [*Wnt1-Cre* [Tg(Wnt1-GAL4)11Rth/J Jackson Labs Stock #003829]. This line uses an enhancer of the Wnt1 gene to express Cre in early NCCs^16^. *Nkx2–5*^*IRESCRE*^ [NKx2–5KI^tm2(cre)Rph^/J, Jackson Labs Stock #024637] allele is a knock in of the Cre Recombinase into the Nkx2 homeobox 5 locus, leaving the protein coding region unaltered^17^.Tie2-Cre [*Tg(Tek-cre)1Ywa/J* Jackson Labs #008863]^18^ expresses Cre under the control of the tyrosine kinase Tek^18^. *Nfatc1-Cre* was developed with an insertion of Cre downstream of the mouse *Nfatc1* stop codon^19^. Cre expression was seen in endocardial and in the mesenchymal cells (from the daughter cells of endocardial cells) of the developing heart^19^, this line was kindly provided by Bin Zhou, Einstein College of Medicine, NY^19^. *Mef2c-AHF-Cre*, a transgenic line using Mef2c promoter elements, was kindly provided by B. Black (UCSF, CA)^20^.

### Genotyping

Genotyping was performed using GoTaq^TM^ Hot Start Polymerase: Green Master Mix, 2× (Promega, PRM5122) and Dimethyl Sulfoxide (DMSO) (Sigma, D2650). PCRs were performed on SimpliAmp^TM^ Thermal Cycler (Fisher Scientific, A24811). Primer sequences are listed in Supplementary Table 2.

### Ultrasound assessment of cardiac structure

In utero fetal echocardiography was performed with the Visualsonics Vevo 2100 ultrasound system^8^. Our ultrasound phenotyping pipeline utilized a standard combination of two-dimentional, color flow and spectral Doppler imaging. This was conducted from E13.5 to E15.5, spanning the time when OFT septation and cardiac chamber formation are completed^8^.

### ECM histopathology and volumetric analysis

Fetuses identified with CHD by ultrasound screening were harvested and embedded in paraffin for serial histological imaging using ECM^9^. The serial image stacks were digitally sectioned in different imaging planes and reconstructed in three-dimensions (3D) for CHD diagnosis. DORV is defined as both great arteries arising from the RV with aorta >50% over the RV and/or with aortic-mitral valve discontinuity. 3D Slicer was used to segment and quantify the endocardial and OFT cushions from reconstructions of the serial image stacks from E10.5 samples.

### Sodium dodecyl sulfate (SDS) polyacrylamide gel electrophoresis and immunoblotting

Mouse tissues/cells were lysed in lysis buffer (Lysis Buffer 25 mM Tris-HCl (pH 7.4), 150 mM NaCl 5 mM EDTA, 0.5% w/v TritonX-100 and 50 µM phenylmethylsulfonyl fluoride and complete mini EDTA free protease inhibitor (Roche-Sigma 11836170001). Proteins were quantified using bicinchoninic acid (Pierce) or Bradford (BioRad). Samples with 30 μg of total protein were boiled in SDS sample buffer. SDS denatured samples were resolved on gels prepared from a 30:.04 acrylamide-bis-acrylamide. Gels were transferred on PVDF membrane. Membranes were blocked in Odyssey blocking buffer (PBS) (LI-COR) for 1 h at room temperature. Blocked polyvinylidene fluoride (PVDF) membranes were incubated with antibodies in odyssey blocking buffer in PBST at room temp for 2 h. After washing in PBST, secondary antibody combinations were IRdye 800CW donkey anti-rabbit IgG (1:10,000) and IRDye 680RD donkey anti-mouse IgG (1:10,000) (LI-COR) in PBST and 0.01% SDS for 1 h in dark. Immunoblots were washed four times in PBST with a final wash in PBS. Dried immunoblots were scanned in IR Odyssey imager-classic (LI-COR).

### Whole mount β-galactosidase staining

Embryos were dissected in phosphate-buffered saline (PBS) and fixed with 4% paraformaldehyde in PBS. For detection of β-galactosidase activity, embryos were stained overnight at 37 °C in standard staining solution containing X-gal.

### Analysis of cell proliferation and apoptosis

Embryos embedded in paraffin were sectioned and stained using antibodies to anti-phosphorylated histone H3 antibody (pH3) for assessing cell proliferation^21^ and deoxynucleotidyl TUNEL with a Roche TUNEL assay kit to measure apoptosis^22^.

### Immunohistochemistry and confocal microscopy

Cells or embryos were fixed in either 4% paraformaldehyde or −20 °C methanol. Antibody incubation was carried at room temperature for an hour with cells and overnight at 4 °C for tissue sections using different primary antibodies (Supplementary Table 3). Incubation of secondary antibodies (Supplementary Table 4) were carried out at room temperature for one hour. All immunohistochemistry results are from at least three mutants and three controls. Imaging was conducted using Leica TCS-SP8 or Olympus fluoview confocal microscope. Images were quantitatively analyzed using ImageJ and/or CellProfiler.

### Wound closure assay

Mouse embryonic fibroblasts (MEFs) were isolated from E12.5 to E13.5 mouse embryos. MEFs were grown to confluency, and then a scratch in the monolayer was made using a 20 µL pipette tip attached to a vacuum line, and time lapse images were obtained at 1, 3, 6, 18, and 24 h using a 20× objective to measure the extent of wound closure. After wound closure, a second scratch was made, and 18 h later, cells were fixed and stained with anti-Giantin antibody to measure the angle between the center of mass of the Golgi apparatus and the center of mass of the nucleus, relative to the edge of the wound. Only cells within 100 microns of the wound edge were chosen for angle measurements, while cells >200 microns from the wound edge were used as non-polarized controls.

### Cushion explant cell migration assay

The endocardial cushion from E10.5 embryos was excised and explanted endocardial side down onto rat tail collagen gel (Enzo Life Sciences) and cultured at 37 °C. Cushions were imaged after attachment to the gel, then at 24 and 48 h post-explant to measure gel invasion by cushion cells using ImageJ.

### Vinculin FA and stability assays

MEF cells were stained for Vinculin (Sigma, 1:400, V9131). Images were processed to increase contrast and quantify size and number of FA. Particles less than 0.45 µm^2^ were excluded from quantification.

Cells were transfected with mEmerald-vinculin (Addgene, 54302) using TransIt-X2 (Mirus Biosciences) following manufacturer′s directions. Totally, 48–72 h after transfection, the cells were imaged on a Leica SP8 confocal microscope in resonant scanning mode. A 40×/1.3NA oil immersion objective was used for imaging and adaptive focus control was used to maintain the focal plane during acquisition. To separate FA from cellular background, a narrow pinhole (1 AU) was used and the focal plane set as low as possible. Images were collected every 45 s for 70 min using low laser power (<1%) to prevent photodamage.

To measure the stability of FA, cells were pre-processed using Fiji software (Gaussian blurring to reduce noise, followed by background subtraction). FA were then segmented by intensity thresholding, and a single intensity threshold was used to segment FA at all subsequent time points. The % of segmented pixels overlapping with segmented FA at time 0 was measured for each time point, then the overlap % was graphed vs. time and a mono-exponential decay curve was fit to the plots using Prism 8 (GraphPad, Inc.). A greater mono-exponential decay kinetic (min^−1^) indicates faster turnover of FA.

### Quantitative PCR array

Changes in expression of EMT-related genes were analyzed using a RT^2^ Profiler Mouse EMT PCR Array (PAMM-090Z; QIAGEN). Total RNA of E10.5 endocardial cushion tissue was isolated using RNeasy Plus kit (QIAGEN), then cDNA was synthesized and amplified using Ovation RNA-Esq system V2 kit (TECAN, CA). Amplification and real-time analysis were performed with ABI 7900HT. After normalizing transcript levels to *Gapdh*, relative mRNA levels in control vs. mutant cushions were calculated according to the comparative C_t_ (ddC_t_) method. Cardiac cushions from five pairs of mutants and controls at E10.5 were run on five independent microarrays. Due to the small size of endocardial cushion at E10.5, high variability was expected in transcript measurements. Thus, this assay was run several times to improve the experiment′s signal to noise ratio. In each run, samples with unacceptably low copy number (raw Ct > 40) were excluded for quality control purposes. Then the ddCt values for each transcript were filtered with an outlier test (ROUT, *Q* = 10%) in Prism 8 (Graphpad Inc.), and column statistics were performed on remaining ddCt values using a one-sample *t* test against a hypothetical value of 0. Two types of transcript were identified: strongly responsive genes (*p* < 0.05), and weakly responsive genes (*p* ≥ 0.05, but the mean > variance). These genes were then entered into IPA software (Qiagen, Inc.) for pathway analysis.

### Statistics and reproducibility

Experiments were performed using multiple wildtype and mutant embryos from the same litter. The results generated from mutants from the same litter were averaged, with the same done for wildtype embryos. Then, experiments were replicated with more litters, with each litter representing an *n* of 1. Multiple litters were used for all statistics. For experiments comparing wild-type and mutants, statistical comparison was performed using unpaired two-way Student's *t* test for most and Morpheus (Broad Institute) and one-sample two-tailed *t* test for EMT-related gene expression comparisons.

### Reporting summary

Further information on experimental design is available in the Nature Research Reporting Summary linked to this paper.

## Supplementary information

Supplementary Information

Description of Additional Supplementary Items

Supplementary Movie

Reporting Summary

## Data Availability

The authors declare that all data supporting the findings of this study are available within the paper
