# Supplementary material for: Mutation of LRP1 in cardiac neural crest cells causes congenital heart defects by perturbing outflow lengthening
Source: Commun Biol. 2020 Jun 16;3:312. doi: 10.1038/s42003-020-1035-9 (PMC7297812; doi:10.1038/s42003-020-1035-9)
Supplement: Supplementary file 1 — Supplementary Information [file 42003_2020_1035_MOESM1_ESM.pdf]

Supplementary Information

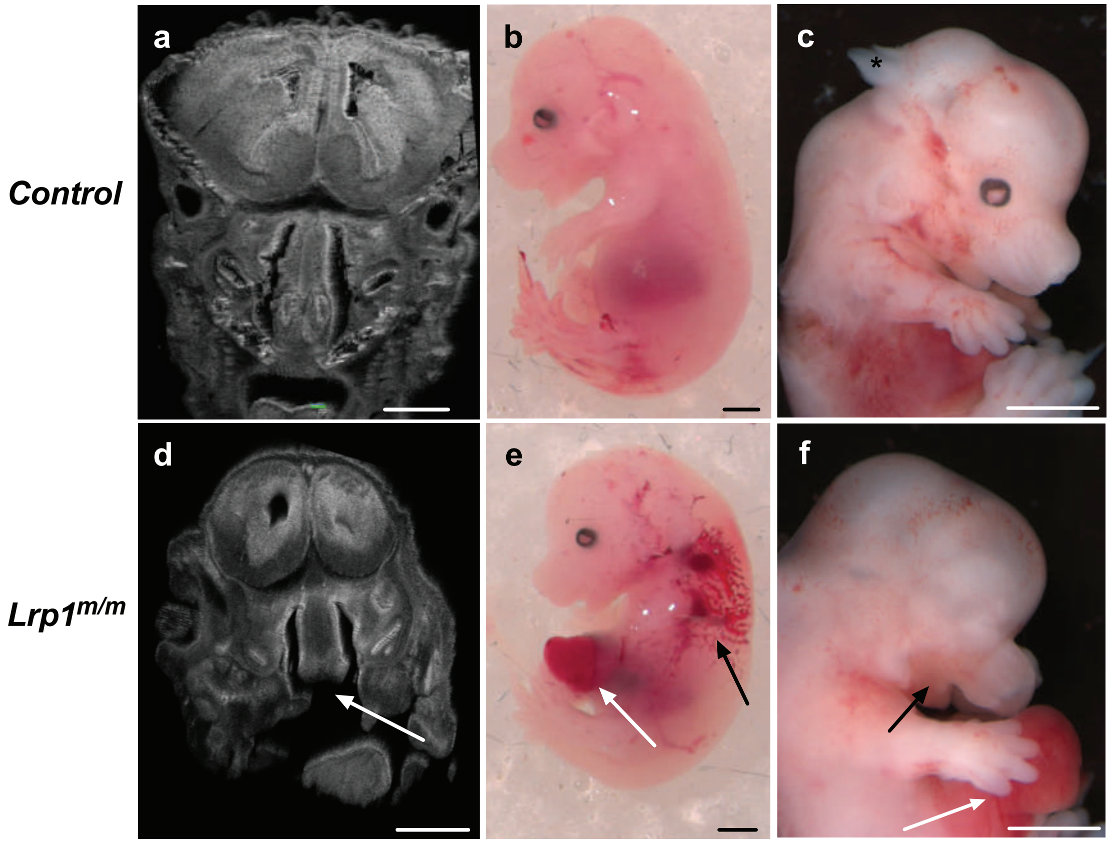

Supplementary Fig 1. Extra-cardiac phenotype of *Lrp1*$^{m/m}$ mutants. Compared with control(a, b c) littermate, *Lrp1*$^{m/m}$ mutant (d, e, f) demonstrated central complete cleft palate (arrow in d), telangiectasia (black arrow in e), gastroschisis (white arrow in e, f) and micrognathia (black arrow in f). Gastroschisis – liver was protruding outside the abdominal cavity through a hole in the abdominal wall. The protruding liver was not covered in a membrane (white arrow in e and f). Scale bars: 0.5mm in a and d; 1mm in b, e, f.

a

Cardiac Phenotypes of *Lrp1*$^{m/m}$

Cardiac phenotypes N=32	DORV+AVSD 90.63% (N=29)	DORV 6.25% (N=2)	AVSD 3.12% (N=1)
Unbalanced AVSD (LV dominant)	34.5% (N=10)	0	100% (N=1)
IAA type B	10.3% (N=3)	0	0
Hypoplastic transverse arch	6.9% (N=2)	0	0
Right arch	3.5% (N=1)	0	0

b

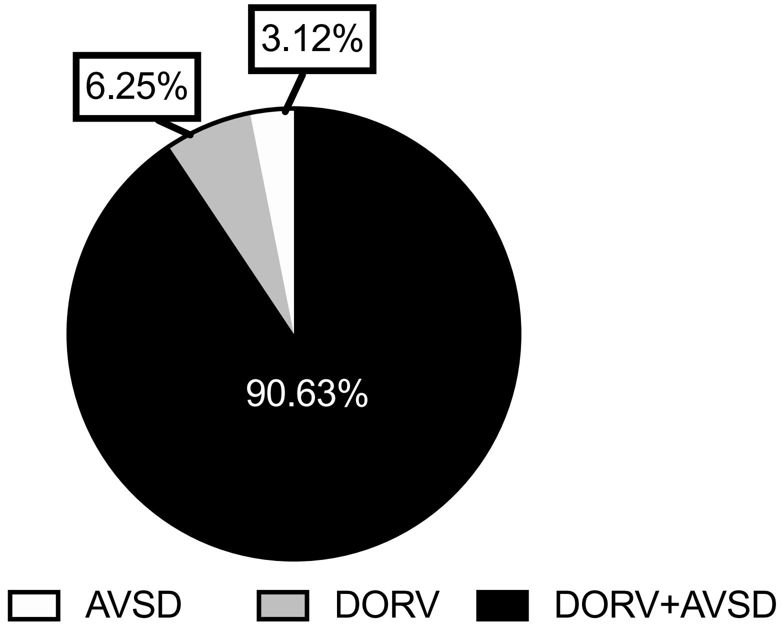

Supplementary Fig 2.(a) Table 1 demonstrates the distribution of different cardiac phenotype o f *Lrp1*$^{m/m}$ mutants. (b) Pie chart of the distribution of cardiac phenotype of *Lrp1*$^{m/m}$ mutants with the majority of mutants exhibiting DORV and AVSD.

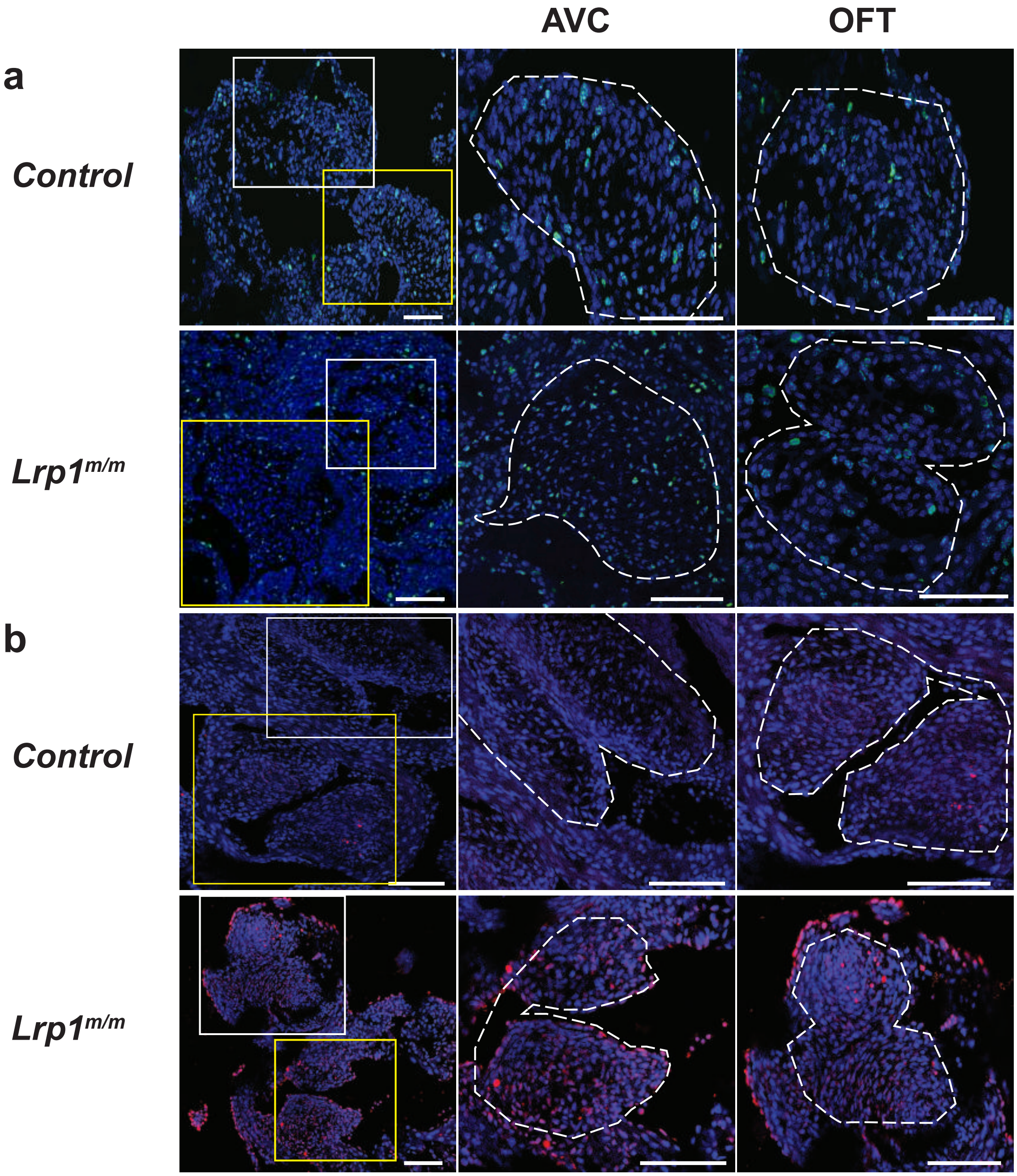

Supplementary Fig 3.(a) Cell proliferation rate was determined using the ratio of positive PH 3 cells/total cushion cells in AVC and OFT cushion PH3 staining. (b) TUNEL assay shows apoptotic cells (red) in atrioventricular cushion and outflow tract cushion of E10.5 control (*Lrp1*$^{+/+}$) and *Lrp1*$^{m/m}$ mutants. The magnified pictures of AVC are from the white box from the right panel and the magnified pictures of OFT are from the yellow box from the right panel. Scale bars: 100 μm.

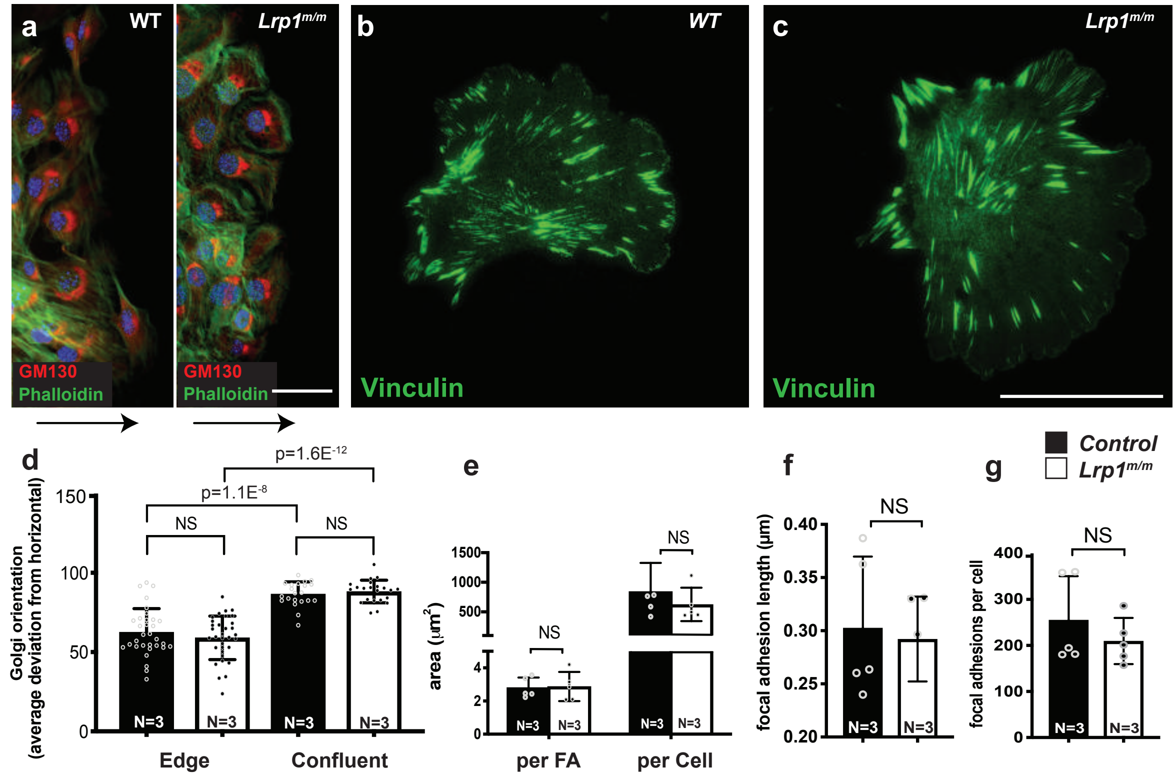

Supplementary Fig 4. (a) Analysis of the orientation of the Golgi labeled with anti-Giantin antibody (red) and phalloidin (green). (d) Quantitative measurement of the Golgi orientation demonstrated no difference between the control and *Lrp1*$^{m/m}$ mutant, neither close to the wound edge nor away from the wound edge in confluent areas. (b,c) MEF cells were stained for Vinculin, and images were processed to increase contrast and quantify size and number of focal adhesions. Particles less than 0.45 μm^2 were excluded from quantification. (e,f, g) Quantitative measurement demonstrated no difference between control and *Lrp1*$^{m/m}$ mutant in the area per focal adhesion, area per cell, length of focal adhesion and number of focal adhesions per cell. Scale bars: 50μm.

Supplementary Table 1. 10 homozygous coding mutations were identified from line 1554

Gene	Chr	Mutation Position	Ref	Alt	Mutation
Ccdc53	10	87664630	A	G	c.A125G:p.N42S
Ern2	7	129321282	C	T	c.G928A:p.V310M
Hsp90aa1	12	111930377	T	C	c.A2039G:p.E680G
Lrp1	10	126978069	A	G	c.T12694C:p.C4232R
Mettl9	7	128195689	C	A	c.C445A:p.L149I
Rfx6	10	51431681	T	A	c.T48A:p.N16K
Tbc1d12	19	38957237	C	T	c.C953T:p.T318I
Trhde	10	114004196	A	G	c.T1577C:p.I526T
Trmt5	12	74383712	C	A	c.G658T:p.V220F
Zfp784	7	4987997	C	T	c.G163A:p.V55I

Supplementary Table 2. PCR primers used to confirm genotypes

Name	Oligonucleotide Sequences (Forward & Reverse)
LRP1 Flox genotype primers	F: CATACCCTCTTCAAACCCCTTCCTG
	R: GCAAGCTCTCCTGCTCAGACCTGGA
1554 LRP1 genotype primers	F: AGATGGGGTTGAAGTGGATG
	R: CCTTCCCATGCTCTGATGA
Universal Cre Recombinase genotype primers	F: GGACATGTTCAGGGATCGCCAGGCG
	R: GCATAACCAGTGAAACAGCATTGCTG
Nkx2.5 Cre specific genotype primers	Wildtype F: GAGCCTGGTAGGGAAAGAGC
	Mutant F: TTACGGCGCTAAGGATGACT
	R: GTGTGGAATCCGTCGAAAGT
Tie2 Cre specific genotype primers	F: CCCTGTGCTCAGACAGAAATGAGA
	R: CGCATAACCAGTGAAACAGCATTGC

Supplementary Table 3. Primary Antibodies

Primary Antibody	Type	Dilution	Manufacturer	Catalog Number
Anti-AP-2 α	Mouse IgG2b Monoclonal	1:50	Developmental Studies Hybridoma Bank	3B5
Anti-CD31	Rat IgG2a Monoclonal	1:250	BD Biosciences	557355
Anti-Clathrin Heavy Chain	Mouse IgG1 Monoclonal	1:250	Sigma Aldrich	C1860
Anti-EEA1	Mouse IgG2a Monoclonal	1:250	Sigma Aldrich	E7659
Anti-GM130	Mouse IgG1 Monoclonal	1:250	BD Transduction Laboratories	51-9001978
Anti-Islet1	Mouse IgG1 Monoclonal	1:25	Developmental Studies Hybridoma Bank	40.3A4
Anti-KDEL	Rat Monoclonal	1:250	Abcam	ab50601
Anti-Ki67	Rabbit Polyclonal	1:500	Abcam	ab15580
Anti-LRP1	Rabbit Monoclonal	1:500	Abcam	ab92544
Anti-NFATc	Mouse IgG1 Monoclonal	1:50	Developmental Studies Hybridoma Bank	7A6
Anti-Periostin	Rabbit Polyclonal	1:250	Abcam	ab14041
Anti-Phospho-Histone H3	Mouse Monoclonal	1:500	Abcam	ab14955
Anti-Vinculin	Mouse IgG1 Monoclonal	1:400	Sigma	V9131
DAPI		1:100	Thermo Scientific	62248

Supplementary Table 4. Secondary Antibodies

Secondary Antibody	Type	Dilution	Manufacturer	Catalog Number
Donkey anti-mouse Alexa Flour 488	IgG(H+L)	1:1000	Invitrogen	A-21202
Goat anti-mouse Alexa Flour 647	IgG2a	1:1000	Invitrogen	A-21241
Donkey anti-rabbit Alexa Flour 555	Rabbit	1:1000	Invitrogen	A-31572
Goat anti-Mouse Alexa Flour 647	IgG1	1:1000	Fisher Scientific	A-21141
Goat anti-Mouse Alexa Flour 488	IgG2b	1:1000	Fisher Scientific	A-21141
Donkey anti-Rat Alexa Flour 488	IgG(H+L)	1:1000	Fisher Scientific	A-21208
