# Supplementary material for: Mutation of LRP1 in cardiac neural crest cells causes congenital heart defects by perturbing outflow lengthening
Source: Commun Biol. 2020 Jun 16;3:312. doi: 10.1038/s42003-020-1035-9 (PMC7297812; doi:10.1038/s42003-020-1035-9)
Supplement: Supplementary file 2 — Description of Additional Supplementary Items [file 42003_2020_1035_MOESM2_ESM.docx]

**Description of Additional Supplementary Files**

Supplementary Movie

Fibroblasts from E12.5-13.4 Lrp1+/+ (wildtype, left) and Lrp1m/m (right) embryos were transiently transfected with vinculin-GFP (Addgene #67935) and imaged using a confocal microscope in a temperature and CO2 controlled incubator. Frame interval, 45s.
