# Supplementary material for: Mutation of LRP1 in cardiac neural crest cells causes congenital heart defects by perturbing outflow lengthening
Source: Commun Biol. 2020 Jun 16;3:312. doi: 10.1038/s42003-020-1035-9 (PMC7297812; doi:10.1038/s42003-020-1035-9)
Supplement: Supplementary file 4 — Reporting Summary [file 42003_2020_1035_MOESM4_ESM.pdf]

nature research

Corresponding author(s): COMMSBIO-19-1553A

Last updated by author(s): May 1, 2020

Reporting Summary

Nature Research wishes to improve the reproducibility of the work that we publish. This form provides structure for consistency and transparency in reporting. For further information on Nature Research policies, see Authors & Referees and the Editorial Policy Checklist .

Statistics

For all statistical analyses, confirm that the following items are present in the figure legend, table legend, main text, or Methods section.

n/a	Confirmed	
☐	☒	The exact sample size (*n*) for each experimental group/condition, given as a discrete number and unit of measurement
☐	☒	A statement on whether measurements were taken from distinct samples or whether the same sample was measured repeatedly
☐	☒	The statistical test(s) used AND whether they are one- or two-sided<br>*Only common tests should be described solely by name; describe more complex techniques in the Methods section.*
☒	☐	A description of all covariates tested
☐	☒	A description of any assumptions or corrections, such as tests of normality and adjustment for multiple comparisons
☐	☒	A full description of the statistical parameters including central tendency (e.g. means) or other basic estimates (e.g. regression coefficient) AND variation (e.g. standard deviation) or associated estimates of uncertainty (e.g. confidence intervals)
☒	☐	For null hypothesis testing, the test statistic (e.g. *F*, *t*, *r*) with confidence intervals, effect sizes, degrees of freedom and *P* value noted<br>*Give P values as exact values whenever suitable.*
☒	☐	For Bayesian analysis, information on the choice of priors and Markov chain Monte Carlo settings
☒	☐	For hierarchical and complex designs, identification of the appropriate level for tests and full reporting of outcomes
☐	☒	Estimates of effect sizes (e.g. Cohen's *d*, Pearson's *r*), indicating how they were calculated

Our web collection on statistics for biologists contains articles on many of the points above.

Software and code

Policy information about availability of computer code

Data collection	Leica LAS-X v3.1.5 and v3.0.3; Leica LAS-AF v2.3.1; ProgRes CapturePro Mac v2.7.6; Micro-Manager v1.4
Data analysis	Fiji v2; GraphPad Prism v8; ; Excel v16; SVI Huygens Essential v16.10; Slicer v4.10; CellProfiler v3.0

For manuscripts utilizing custom algorithms or software that are central to the research but not yet described in published literature, software must be made available to editors/reviewers. We strongly encourage code deposition in a community repository (e.g. GitHub). See the Nature Research guidelines for submitting code & software for further information.

Data

Policy information about availability of data

All manuscripts must include a data availability statement. This statement should provide the following information, where applicable:

- Accession codes, unique identifiers, or web links for publicly available datasets
- A list of figures that have associated raw data
- A description of any restrictions on data availability

Source data for figure(s) [number(s)] are provided with the paper.

Field-specific reporting

Please select the one below that is the best fit for your research. If you are not sure, read the appropriate sections before making your selection.

☒ Life sciences ☐ Behavioural & social sciences ☐ Ecological, evolutionary & environmental sciences

For a reference copy of the document with all sections, see nature.com/documents/nr-reporting-summary-flat.pdf

Life sciences study design

All studies must disclose on these points even when the disclosure is negative.

Sample size	n=4 was the standard choice, except when constrained by the availability of animal subjects. Experiments with greater inherent variability, e.g. qPCR using very small amounts of input RNA, were repeated more (n=5) to account for the expected statistical noise.
Data exclusions	In experiments with high statistical noise (see above), a basic outlier test was used to exclude erroneous values
Replication	All replicates of experiments described in this paper are shown or included in statistics, unless excluded by an outlier test (stated in the text).
Randomization	No population statistics were done in this project.
Blinding	Blinding was not done in this project. All experiments were performed with internal controls (i.e., litter-matched wild-type vs. mutant mice) and automated acquisition and analysis were performed to the extent possible in order to remove experimenter biases.

Reporting for specific materials, systems and methods

We require information from authors about some types of materials, experimental systems and methods used in many studies. Here, indicate whether each material, system or method listed is relevant to your study. If you are not sure if a list item applies to your research, read the appropriate section before selecting a response.

Materials & experimental systems

n/a	Involved in the study	
☐	☒	Antibodies
☐	☒	Eukaryotic cell lines
☒	☐	Palaeontology
☐	☒	Animals and other organisms
☒	☐	Human research participants
☒	☐	Clinical data

Methods

n/a	Involved in the study	
☒	☐	ChIP-seq
☒	☐	Flow cytometry
☒	☐	MRI-based neuroimaging

Antibodies

Antibodies used	KDEL (ab50601), GM130, ppH3, LRP1, EEA1, LAMP, AP-2 alpha, CD31,Islet1, vinculin, Nfatc, Periostin
Validation	

Eukaryotic cell lines

Policy information about cell lines

Cell line source(s)	Embryonic fibroblasts derived from mutant mice and wild-type litter-matched controls
Authentication	*Describe the authentication procedures for each cell line used OR declare that none of the cell lines used were authenticated.*
Mycoplasma contamination	Cell lines were tested by careful examination of DAPI-stained cells growing on coverslips
Commonly misidentified lines (See ICLAC register)	*Name any commonly misidentified cell lines used in the study and provide a rationale for their use.*

Animals and other organisms

Policy information about studies involving animals; ARRIVE guidelines recommended for reporting animal research

Laboratory animals	BL6-derived mice were mutagenized with ENU and then back-crossed with BL6 to isolate mutations and phenotypes of interest.
Wild animals	*Provide details on animals observed in or captured in the field; report species, sex and age where possible. Describe how animals were caught and transported and what happened to captive animals after the study (if killed, explain why and describe method; if released, say where and when) OR state that the study did not involve wild animals.*
Field-collected samples	*For laboratory work with field-collected samples, describe all relevant parameters such as housing, maintenance, temperature, photoperiod and end-of-experiment protocol OR state that the study did not involve samples collected from the field.*
Ethics oversight	All experiments were conducted using protocols approved by the university IACUC and IBC committees.

Note that full information on the approval of the study protocol must also be provided in the manuscript.
